# Angioimmunoblastic T‐cell lymphoma: Characterization of clonal T and B cells and a patient‐derived xenograft study of coexisting T‐ and B‐cell proliferation

**DOI:** 10.1002/jha2.1080

**Published:** 2025-01-28

**Authors:** Xiaoxian Zhao, Deepa Jagadeesh, Juraj Bodo, Lisa Durkin, Daniel J. Lindner, Sarah L. Ondrejka, Eric D. Hsi

**Affiliations:** ^1^ Pathology and Lab Medicine Institute Cleveland Clinic Cleveland Ohio USA; ^2^ Wake Forest University School of Medicine Winston Salem North Carolina USA; ^3^ Taussig Cancer Center Cleveland Clinic Cleveland Ohio USA; ^4^ Present address: Department of Laboratory Medicine and Pathology Mayo Clinic Rochester Minnesota USA

**Keywords:** AITL, EBV, PDX model, SLAMF7

## Abstract

**Introduction:**

Angioimmunoblastic T‐cell lymphoma (AITL) is a rare and aggressive lymphoma with a poor prognosis. AITL is associated with Epstein–Barr virus (EBV)‐positive B cells in most cases, suggesting a possible role for the virus in the pathobiology of AITL. Cell lines from AITL patients do not exist and models of human AITL are needed. We aim to establish such a model and use it for preclinical therapeutic evaluation.

**Methods:**

Primary lymph node tissue from an AITL patient was used for tumor cell isolation and injection to NSG mice. The established patient‐derived xenograft (PDX) model was characterized by immunophenotyping, whole‐exome sequencing (WES), and T/B‐cell receptor gene rearrangement studies. In vivo AITL PDX trials were performed with elotuzumab, romidepsin, and rituximab.

**Results:**

An AITL PDX mouse model that includes a coexisting EBV+ B‐cell proliferation was established. We confirmed clonal identity of the engrafted T cells with the primary T‐lymphoma cells. WES on DNA from xenografted sorted T and B cells identified eight and three mutations previously reported in the COSMIC database, respectively. Primary tumor cells could be passaged serially in NSG mice with an increasing percentage of monoclonal B cells that mimic the human condition in which the clonal B‐cell component in some cases may mask an underling T‐cell lymphoma. In this PDX mouse study, single agent elotuzumab or rituximab significantly improved mice survival. Survival was further improved when elotuzumab or romidepsin was combined with rituximab.

**Conclusion:**

To our knowledge, this is the first molecular characterization of AITL model coexisting with associated EBV+ B cells, and use of such a PDX model for therapeutic evaluation of agents targeting both malignant T cells and B cells simultaneously.

## 1 INTRODUCTION

AITL is a rare and aggressive type of lymphoma that accounts for about 20% of peripheral T‐cell lymphomas (PTCLs) with a 5‐year overall survival of 32%–44% [1, 2]. Thus, new therapies are needed. Signaling lymphocytic activation molecule F7 (SLAMF7), a molecule expressed on a subset of T cells, activated B cells, macrophage, and myeloma cells [3], is an attractive target to explore based on studies showing SLAMF7 expression in a subset of AITL cases [4].

The neoplastic AITL cells are derived from mature CD4^+^ follicular helper T(Tfh) cells [5, 6, 7, 8]. Infiltration of accessory cells contributes to the tumor microenvironment of AITL. One characteristic feature of AITL is that Epstein–Barr virus (EBV)‐positive B cells are detectable in the majority of cases [9, 10, 11]. EBV reactivation may be a consequence of host immune dysfunction. However, the EBV‐positive B cells may progress, either as a composite lymphoproliferative process or an overt EBV‐associated large B‐cell lymphoma independent of the T‐cell lymphoma [12, 13, 14]. Next‐generation sequencing (NGS) studies have identified recurrent somatic mutations in AITL, and demonstrated common mutations in epigenetic pathway genes such as *TET2* and *DNMT3A* in hematopoietic stem cells and AITL cells [15]. These are followed by additional mutations in genes such as *RHOA* that induce Tfh specification and promote lymphomagenesis [16]. Experimental models for AITL that also recapitulate the common occurrence of coexisting EBV+ B‐cell lymphoproliferative processes are lacking and thus, potential interactions between the two are largely unexplored.

We report a patient‐derived xenograft (PDX) study of coexisting AITL and EBV+ B‐cell proliferations in which the T‐cell component appeared reliant on the presence of the B‐cell component. We characterized this model at the phenotypic and genotypic levels and evaluated the in vivo efficacy of romidepsin, elotuzumab, rituximab alone or in combination in a preclinical model.

## 2 MATERIALS AND METHODS

### 2.1 Patient sample

Primary lymph node tissue was collected from an AITL patient with Institutional Review Board approval. Fresh tissue was finely diced and passed through a 40 µM cell strainer. Isolated cells were kept in RPMI1640 with 10% fetal bovine serum.

### 2.2 PDX murine model of AITL

Experimental procedures were performed in the animal research core of Lerner Research Institute and were approved by the Institutional Animal Care and Use Committee. Six‐ to 8‐week‐old NSG (NOD‐SCID‐IL‐2rγ null) mice were irradiated (2.25 Gy) 1 day before inoculation of 1 × 10^7^ cells by tail vein. Mouse blood was collected for flow cytometric assay of engrafted human tumor cells and quantitated by human CD45, CD3, and CD20 expression. Mice were euthanized when mice demonstrated greater than 20% weight loss, lethargy, or other generalized symptoms. Organs were collected at time of euthanasia. Tumor cells were serially passaged in NSG mice by tail‐vein inoculation of isolated cells from engrafted mouse tissues.

### 2.3 Immunophenotyping, immunohistochemistry, and CISH‐EBER, enrichment/sorting of engrafted tumor cells and DNA extraction, T‐ and B‐cell clonality analysis, whole‐exome sequencing

Detailed methods are in Supporting Information

### 2.4 In vivo AITL PDX trial

Tumor cells collected from second passage of the AITL‐engrafted mouse were inoculated intravenously (i.v.) via tail vein into NSG mice. When engraftment in peripheral blood was detectable 3 weeks later, mice were randomly assigned to six groups with eight mice per group for treatment in following cohorts: (I) intraperitoneal injection of romidepsin with dosing of 1 mg/kg, three times per week and continued during the study; (II) one‐time intravenous injection of rituximab at dosing of 20 mg/kg; (III) intraperitoneal injection of elotuzumab at dosing of 5 mg/kg, three times per week for 3 weeks; (IV) romidepsin plus rituximab; (V) elotuzumab plus rituximab; and (VI) vehicle control. All mice were ear tagged and monitored. Mice were euthanized when they met experimental endpoint (>20% weight loss, lethargy, other generalized symptoms). The Cox's *F* test in the Kaplan–Meier survival analysis for two groups was used. *p‐*Value of less than 0.05 was considered significant.

## 3 RESULTS

### 3.1 Primary AITL PDX in NSG mice

An axillary lymph node was biopsied from a 53‐year‐old woman, and diagnosis of AITL was rendered. EBER‐ISH was negative. The patient was treated with six cycles of CHOEP followed by autologous stem cell transplantation. Three months after transplantation (9 months after diagnosis), the patient developed progressive fatigue and arthralgias. PET‐CT scan showed new cervical, thoracic, abdominal, and pelvic lymphadenopathy.

A cervical lymph node biopsy confirmed AITL relapse. Immunohistochemistry (IHC) staining showed the atypical cells were T cells expressing CD2, CD3, CD4, CD5, CD7, CD10, BCL6, PD1, and CXCL13, while negative for CD8, CD20, CD30, ALK‐1, TIA‐1, granzyme B, and TCL1A. CD21 stain highlighted abnormal follicular dendritic cell proliferations. Scattered CD20^+^ B cells including B‐immunoblasts were present with focal large clusters/small sheets. EBER‐ISH was positive in scattered large immunoblasts. Focal large clusters/small sheets of EBV+ large cells were also present, which were not present prior to initial chemotherapy.

**FIGURE 1:**
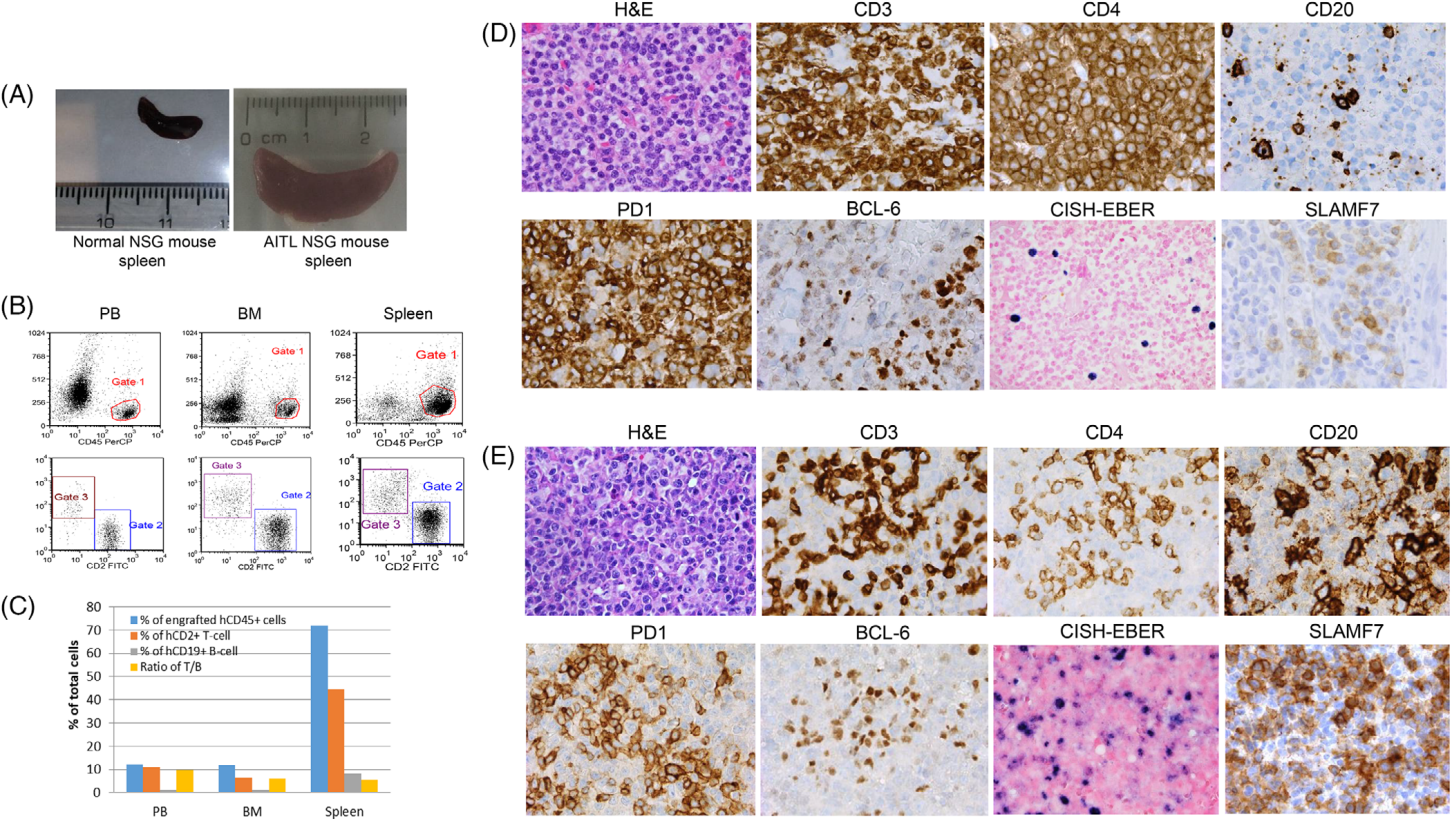
Engraftment of primary AITL cells in NSG mouse. (A) Gross image of harvested spleens. The AITL engrafted spleen is markedly enlarged compared to the control spleen. (B) Flow cytometry of the passage 1 (P1) engrafted mouse tissues. Human CD45 expressing cells are present in blood, bone marrow, and spleen consisting of predominantly T cells (gate 2) with fewer B cells (gate 3). (C) The percentage of human T and B cells in various sites of the P1 mouse. A predominance of T cells was seen. (D) The primary relapsed lymph node showed atypical Tfh phenotype T cells (CD4^+^/PD1^+^/BCL6^+^) with scattered large EBV+ B cells. Note the large immunoblastic morphology by CD20 staining. SLAMF7 is expressed in a subset of cells. (E) IHC of the engrafted P1 spleen shows mixed lymphomatous T cells expressing CD3, CD4, PD1, and BCL6. Numerous EBV+ large cells are present. SLAMF7 is seen in many of the small to intermediately sized cells compatible with the T cells.

Primary cells isolated from the cervical lymph node were (i) cultured ex vivo but failed to proliferate; and (ii) inoculated into one NSG mouse i.v. Engraftment of the primary cells became detectable in peripheral blood after 2 weeks, and the percentage of engrafted cells increased progressively. The mouse was euthanized on Day 41 due to weight loss. All mouse organs appeared normal in shape and size except for marked splenomegaly (Figure 1A). Flow cytometry assay showed the engrafted cells accounted for 12.3%, 13.1%, and 73.2% of total populations for peripheral blood, bone marrow, and spleen tissue, respectively. CD2^+^/CD19^−^ T cells dominated the engrafted cells in all three samples, while CD19^+^/CD2^−^ B cells were about 8%–12% (Figure 1B,C). Histologic examination of the mouse spleen showed infiltration with malignant cells (designated as first passage P1 cells), and IHC results confirmed presence of human T cells expressing CD3, CD4, PD1, BCL‐6, and SLAMF7, as well as B cells expressing CD20 and EBER in the engrafted mouse tissue, consistent with the primary sample (Figure 1D,E).  

Given the successful engraftment of this AITL with coexisting T‐ and B‐cell proliferations, we tested whether immunomagnetically enriched T cells (ET) could be passaged alone. Five million human lymphoma T cells from the P1 mouse spleen were inoculated into an NSG mouse (ET‐P2). In parallel, another mouse received isolated P1 spleen cells (consisting of neoplastic T and B cells) without subset enrichment (passage 2: P2). Both P2 and ET‐P2 mice were euthanized when they met experiment endpoint at Week 6. The P2 mouse had splenomegaly and enlarged kidney, while the ET‐P2 mouse had no obvious change in organs size. H&E stains showed both these secondary passage mice had infiltration of malignant cells in spleen, kidney, and liver. However, fewer tumor cells were detected in kidney and liver of the ET‐P2 mouse compared to those of the P2 mouse (Figure S1). IHC staining for CD3, PD1, and CD20 confirmed the engraftment of both T and B cells in spleen, kidney, and liver of the P2 mouse, while human T cells, but not B cells, were detected in ET‐P2 mouse organs (Figure S2). IHC also detected SLAMF7^+^ cells in ET‐P2 mouse spleen, which contained numerous CD3^+^ lymphoma cells and lacked B cells by CD20 and EBER‐ISH staining (Figure S3). However, repeated attempts to re‐engraft these ET‐P2 splenic T cells into NSG mice failed with no human T cells detected in blood samples up to 8 months after i.v. inoculation, and those mice remained healthy throughout. In contrast, cells from P2 mouse were capable of further engraftment in NSG mice (passage 3: P3) and reached experimental endpoint (moribund mouse necessitating sacrifice) around Week 6. IHC for CD20, CD3, and CD4 showed an increased proportion of B cells in P3 mice compared to P1 and P2 samples (Figure S4), mimicking the situation in which the B‐cell component might overtake/outgrow the T‐cell lymphoma in humans. Double staining of CD20 and EBER showed co‐staining of these two targets (Figure S5). Double IHC staining showed a subset of T cells co‐expressed SLAMF7 in the engrafted tumor (Figure S6), without detectable CD20/SLAMF7 dual‐positive cells.

### 3.2 Molecular characterization of engrafted T and B cells

Harvested P1 tumor cells from engrafted splenic tissue were sorted into T‐ and B‐cell components with purities of 99.6% and 96.2%, respectively (Figure S7A). Genomic DNA was extracted from the sorted cells. T‐cell receptor gamma (*TCRG*) PCR clonality studies (BIOMED2) confirmed monoclonality of the sorted T cells and matched the pattern obtained from the patient relapsed biopsy (Figure S7B). *IGH* and *IGK* clonality assays (BIOMED2) on DNA from the sorted B cells showed monoclonal rearrangement. No monoclonal B‐cell population was detected in the primary AITL sample of the relapsed biopsy (Figure S7C), likely due to insufficient EBV+ B cells present. These data supported that the engrafted T cells were from the primary malignant AITL cells. However, an EBV+ B‐cell clone was selected during passaging and eventually dominated the B‐cell population in vivo.

Whole‐exome sequencing (WES) was performed on genomic DNA of P1 sorted T and B cells. DNA from sorted patient CD15^+^ peripheral blood neutrophils was used as paired normal control. A total of 33 mutations (31 genes) were private to the T cells only (Table 1), seven of which were reported in COSMIC ( https://cancer.sanger.ac.uk/cosmic ), which includes *RhoA* G17V, one *TET2* splicing mutation, one *TET2* stopgain mutation, two *STAT3* missense mutations of K658N and K658R, and one *VAV1* missense mutation of I803N. Nine mutations were found only in the sorted EBV+ B cells, two of which were reported in COSMIC but are not associated with lymphoma (Table 2). *PRB4* was the only shared mutation in both T and B cells, and was present at relatively low variant allele frequencies (Table 3). While the mutation was reported in COSMIC in carcinoma and melanoma, it is not a driver gene in lymphoma.

**TABLE 1:** Mutants in sorted T cells.

VAF_Ctl	VAF_B	VAF_T	Chr	CytoBand	Func.refGene	Gene	Mutation	RefSeq/DNA change/amino acid change	COSMIC genomic mutation ID
1.0%	0.0%	38.6%	chr3	3q12.2	Exonic	ABI3BP	Nonsynonymous SNV	NM_015429:c.A1516G:p.T506A	No
0.0%	1.0%	24.0%	chr10	10q26.13	Exonic	CHST15	Nonsynonymous SNV	NM_001270765:c.C1358G:p.P453R	No
0.0%	0.0%	29.5%	chr12	12p13.31	Splicing	CLSTN3	Unknown	NM_014718:exon8:c.1323+1G>A	No
0.0%	0.0%	34.1%	chrX	Xq22.3	Exonic	COL4A5	Nonsynonymous SNV	NM_000495:c.G2363A:p.R788H	COSV60369705
0.0%	0.7%	18.6%	chr17	17q23.1	Exonic	DHX40	Nonsynonymous SNV	NM_001166301:c.G1532A:p.R511H	No
0.0%	0.4%	29.5%	chr4	4q35.2	Exonic	F11	Nonsynonymous SNV	NM_000128:c.G1030C:p.G344R	No
0.0%	0.6%	22.5%	chr14	14q22.3	Exonic	FBXO34	Nonsynonymous SNV	NM_017943:c.G652T:p.A218S	No
0.0%	0.0%	29.1%	chr6	6q25.3	Exonic	FNDC1	Frameshift deletion	NM_032532:c.3412delC:p.P1138fs	No
0.0%	0.0%	27.4%	chr8	8p11.23	Exonic	GOT1L1	Nonsynonymous SNV	NM_152413:c.T631A:p.F211I	No
0.0%	1.7%	26.6%	chr4	4p16.1	Exonic	JAKMIP1	Nonsynonymous SNV	NM_001099433:c.C361T:p.R121C	COSV51522294
0.0%	0.7%	29.7%	chr7	7q21.11	Exonic	MAGI2	Nonsynonymous SNV	NM_012301:c.C2288T:p.T763I	No
0.0%	0.0%	29.8%	chr11	11p11.2	Exonic	MAPK8IP1	Nonsynonymous SNV	NM_005456:c.T1700C:p.F567S	No
0.0%	1.4%	28.0%	chr12	12q21.31	Exonic	MGAT4C	Nonsynonymous SNV	NM_013244:c.C13A:p.H5N	No
0.0%	0.0%	38.2%	chr1	1q22	Exonic	PAQR6	Nonsynonymous SNV	NM_001272109:c.C310T:p.H104Y	No
0.0%	7.1%	33.3%	chr5	5q31.3	Exonic	PCDHB13	Nonsynonymous SNV	NM_018933:c.C1693A:p.L565M	No
0.0%	0.0%	40.8%	chr12	12p13.31	Exonic	PEX5	Nonsynonymous SNV	NM_000319:c.C698G:p.A233G	No
0.0%	0.0%	25.8%	chr22	22q13.1	Exonic	PLA2G6	Nonsynonymous SNV	NM_001004426:c.G1614C:p.Q538H	No
0.0%	0.0%	27.7%	chr10	10p12.31	Exonic	PLXDC2	Nonsynonymous SNV	NM_001282736:c.A209G:p.D70G	No
0.8%	0.0%	34.0%	chr3	3p21.31	Exonic	RHOA	Nonsynonymous SNV	NM_001664:c.G50T:p.G17V	COSV69041529
0.0%	0.0%	32.8%	chr3	3p21.2	Exonic	RRP9	Nonsynonymous SNV	NM_004704:c.G1309C:p.V437L	No
0.0%	2.9%	14.3%	chr22	22q12.2	Exonic	SFI1	Nonsynonymous SNV	NM_001258325:c.C1866A:p.S622R	No
0.0%	0.0%	18.7%	chr2	2q24.2	Exonic	SLC4A10	Nonsynonymous SNV	NM_022058:c.C2998T:p.R1000W	No
0.0%	0.5%	23.7%	chr17	17q21.2	Exonic	STAT3	Nonsynonymous SNV	NM_003150:c.G1974C:p.K658N	COSV52895397
0.0%	0.0%	22.8%	chr17	17q21.2	Exonic	STAT3	Nonsynonymous SNV	NM_003150:c.A1973G:p.K658R	COSV52886492
0.0%	0.9%	32.8%	chr14	14q12	Exonic	STXBP6	Stopgain	NM_001304477:c.C259T:p.Q87X	No
0.0%	0.7%	35.4%	chr4	4q24	Splicing	TET2	Unknown	NM_001127208:exon9:c.4045‐1G>C	COSV54420035
0.0%	0.7%	33.2%	chr4	4q24	Exonic	TET2	Stopgain	NM_001127208:c.C2797T:p.Q933X	COSV54407557
0.0%	0.0%	26.7%	chr2	2q31.2	Exonic	TTN	Nonsynonymous SNV	NM_133378:c.T10661C:p.L3554P	No
0.0%	2.1%	37.1%	chr6	6q25.3	Exonic	TULP4	Nonsynonymous SNV	NM_020245:c.G3386A:p.G1129D	No
0.0%	0.5%	24.4%	chr2	2q34	Exonic	UNC80	Nonsynonymous SNV	NM_032504:c.G644T:p.W215L	No
0.0%	0.0%	41.9%	chr19	19p13.3	Exonic	VAV1	Nonsynonymous SNV	NM_001258206:c.T2408A:p.I803N	No
1.4%	0.0%	28.1%	chr5	5q13.2	Exonic	ZNF366	Nonsynonymous SNV	NM_152625:c.C445T:p.P149S	No
0.0%	0.0%	28.4%	chr19	19p13.2	Exonic	ZNF440	Frameshift deletion	NM_152357:c.1331delG:p.R444fs	No

**TABLE 2:** Mutants in sorted B cells.

VAF_Ctl	VAF_B	VAF_T	Chr	cytoBand	Func.refGene	Gene	Mutation	RefSeq/DNA change/amino acid change	COSMIC genomic mutation ID
0.0%	34.4%	1.0%	chr1	1q32.1	Exonic	CHI3L1	Nonsynonymous SNV	NM_001276:c.A196T:p.I66F	No
0.0%	41.9%	0.0%	chr2	2q14.2	Exonic	CLASP1	Nonsynonymous SNV	NM_001142273:c.C829T:p.R277W	No
0.0%	37.6%	0.6%	chr19	19q13.43	Exonic	NLRP5	Nonsynonymous SNV	NM_153447:c.C2953A:p.L985M	No
0.0%	36.0%	0.0%	chr4	4p16.1	Exonic	PSAPL1	Nonsynonymous SNV	NM_001085382:c.G1438A:p.D480N	COSV59844480
0.5%	33.9%	0.0%	chr11	11p12	Exonic	RAG2	Stopgain	NM_000536:c.G661T:p.G221X	No
0.0%	35.4%	0.0%	chr2	2q11.2	Exonic	REV1	Nonsynonymous SNV	NM_001037872:c.C2554G:p.R852G	No
0.0%	37.2%	0.0%	chr2	2q37.3	Exonic	SNED1	Nonsynonymous SNV	NM_001080437:c.G1082A:p.G361D	No
0.0%	37.9%	0.0%	chr8	8p12	Exonic	TEX15	Nonsynonymous SNV	NM_031271:c.T6618G:p.N2206K	No
0.0%	41.2%	0.0%	chr4	4q35.2	Exonic	TRIML1	Nonsynonymous SNV	NM_178556:c.C1298T:p.P433L	COSV60193139

**TABLE 3:** Mutants in sorted T and B cells.

VAF_Ctl	VAF_B	VAF_T	Chr	cytoBand	Func.refGene	Gene	Mutation	RefSeq/DNA change/amino acid change	COSMIC genomic mutation ID
2.9%	18.4%	14.0%	chr12	12p13.2	Exonic	PRB4	Nonsynonymous SNV	NM_002723:c.C396A:p.H132Q	COSV54396381

### 3.3 In vivo PDX trial

**FIGURE 2:**
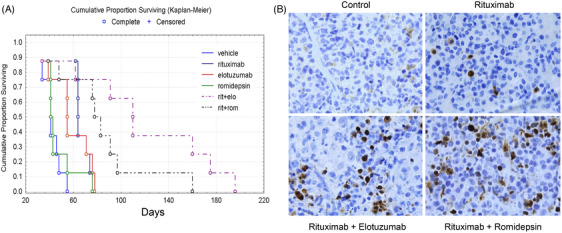
In vivo treatment of AITL PDX mice. (A) Treatment with single agent of rituximab, elotuzumab, and two‐drug combined groups extended survival compared to control supported by statistical analysis (*p* < 0.05). The combination of rituximab with either elotuzumab or romidepsin further prolonged survival compared to each single agent significantly (*p* < 0.05). (B) IHC staining of cleaved PARP in control, and each indicated mouse spleen tissue. No detectable staining in control sample, while cleaved PARP‐positive cells show in rituximab‐treated mouse spleen, and more positive cells presented in mice samples treated with rituximab combined with either elotuzumab or romidepsin.

In a proof‐of‐concept preclinical therapeutic model, we explored potential new therapies in this combined T‐cell and EBV+ B‐cell AITL PDX. We previously showed that SLAMF7 is expressed in a subset of NK/T‐cell lymphomas [3, 17] and demonstrated this case expressed SLAMF7. We thus performed a therapeutic experiment comparing romidepsin (approved use in relapsed/refractory PTCLs), rituximab, elotuzumab (anti‐SLAMF7 antibody approved for use in multiple myeloma), and combinations of these agents. Briefly, we passaged the bulk tumor cells isolated from the P2 mouse spleen (Figure S4) in NSG mice (passage 3). Engraftment was monitored in peripheral blood by flow cytometry. Three weeks after inoculation, mice were assigned to experimental groups to have balanced average engraftment levels in each cohort at initiation of therapy. Figure 2A shows the overall efficacy of each treatment on survival of the PDX mice. Four of five treated cohorts, including single agent, elotuzumab or rituximab and the two combined therapy groups, significantly extended survival compared to control (*p* < 0.05). Romidepsin alone showed no significant survival effect compared to control (*p* = 0.27). There was no significant difference in survival between elotuzumab and rituximab. Combination of rituximab with either elotuzumab or romidepsin significantly improved survival in comparison to each single agent (*p* < 0.05). A trend for prolonged survival for rituximab/elotuzumab compared to rituximab/romidepsin was observed but was not significant (*p* = 0.067). IHC staining of mice spleen tissues for cleaved PARP (a marker of apoptosis) was negative in control group; cleaved PARP was detectable in rituximab‐treated mice samples and more positive cells in two drugs combined groups (Figure 2B). IHC for CD3, CD4, and CD20 showed alterations in the T‐ and B‐cell populations in mouse spleen after different treatment regimens (Figure S8).

## 4 DISCUSSION

PDX mouse models may aid in our understanding of tumor biology, and facilitate preclinical evaluation of novel therapeutic strategies. This assumes the models more closely resemble primary tumors than other in vitro systems. We successfully performed an AITL PDX mouse study with coexisting T‐ and B‐cell compartments and confirmed that engrafted T cells were from the same clone as the original tumor. The primary tumor cells were capable of serial passage in NSG mice with the majority of engrafted cells being malignant T cells in the first passage. Further passages in mice resulted in an increase of monoclonal EBV+ B cells (Figure S4) that mimics the clinical situation in which the B‐cell component might ultimately mask an underlying T‐cell lymphoma.

Because the majority of AITL patients carry EBV+ B cells and the efficacy of treatments specifically targeting B and T cells in patients with significant EBV lymphoid proliferations is not well studied [18], we performed in vivo trials using this PDX model. Specifically, we evaluated romidepsin and two monoclonal antibodies approved for clinical use in other indications. Romidepsin alone did not yield survival benefit. This may reflect the moderate efficacy of romidepsin in relapsed/refractory AITL patients, in which the objective response rate (ORR) and complete response (CR) rate as single agent were 42% (95% confidence interval [CI]: 25%–61%) and 24% (95% CI: 11%–42%), respectively [19]. Rituximab alone extended mouse survival. This could be explained by the significant EBV+ B‐cell component in P3 mice that would be targeted. Successful treatment of an AITL patient with EBV+ B cells using rituximab in combination with chemotherapy has been described [20]. SLAMF7 is expressed in 25%–68% of PTCLs and NK/T‐cell neoplasms [3]. As a single agent, elotuzumab prolonged survival. Further studies with multiparameter flow cytometry or digital spatial profiling would better define SLAMF7 expression in various cell types in AITL. Combination of rituximab with either elotuzumab or romidepsin further significantly improved the survival of mice, associated with the reduction of both engrafted T and B cells compared to control. Our in vivo study suggests that further investigation of elotuzumab is warranted in AITL with an EBV+ component.

Histologically, the AITL microenvironment includes a polymorphic infiltration of malignant cells with an abundance of nonmalignant bystander cells. EBV+ B‐cell proliferations are commonly seen in AITL and can vary from relatively few cells to sheets of cells that can dominate the lesion and obscure the underlying AITL. In this patient, we saw the onset of the EBV+ B‐cell process only at relapse, suggesting initiation of AITL does not require EBV‐infected B‐cells. The focal sheets of EBV+ large B cells in the relapse biopsy are suggestive of early development of an EBV+ large B‐cell lymphoma in the setting of AITL. These were shown to be monoclonal after in vivo expansion in this PDX study, but did not appear to contain typical driver mutations when sorted B cells were studied. This does raise the question of what represents EBV+ lymphoma in the clinical setting. The engrafted EBV+ B cells were shown to be monoclonal but, due to limited cell recovery, we were unable to study whether B‐cell engraftment alone could occur (data not shown). Thus, one could argue the B cells that were being passaged still represented an EBV+ lymphoblastoid cell line rather than overt EBV+ B‐cell lymphoma cells. Nevertheless, the engraftment studies did appear to mimic what occasionally occurs in the clinical setting—namely outgrowth of an EBV+ B‐cell clone capable of independent growth in an AITL patient. Detailed genetic studies of both T‐ and B‐cell components isolated from primary biopsies over time in the same patient would be illuminating to explore independent B‐cell clonal evolution.

Gene expression profiling suggested that normal Tfh cells are the origin of AITL [21]. Within the germinal center, B cells are important for maintenance of Tfh cells, and the frequency of Tfh and B cells correlated positively, suggesting an interdependent relationship [22, 23]. In this study, the enriched AITL T cells were capable of survival and expansion in mice for only one generation and failed to engraft further, while mixed populations of T and B cells could be passaged serially. This suggests that AITL tumor cells may be supported by the B‐cell fraction. We cannot exclude that this phenomenon is an artifact of the PDX process, where T cells might have become more dependent on B cells. However, the observed apparent dependence would be consistent with recent murine model data suggesting B cells in AITL, perhaps derived from a precursor stem cell with a similar genetic background, may clonally evolve and provide a supportive niche [24]. Whether the EBV+ B cells contributed to the relapse in this patient is unclear, but interesting to consider. In select cases targeting B cells or specific B‐cell‐dependent pathways might then be helpful in eliminating a supportive environment. While addition of rituximab to the CHOP did not prove efficacious in overall in AITL [25], a recent study reported that a rituximab and lenalidomide plus chidamide regime may be an effective combination for patients with relapsed/refractory AITL [26].

Detailed genomic analysis of both AITL T‐cell lymphoma and concomitant B‐cell proliferations in a single patient is limited [27]. To our knowledge, this is the first study to compare the AITL T cells and associated EBV+ B cells for any shared mutants, which may be derived from clonal hematopoiesis. Although the recurrent mutations associated with AITL were identified in T cells, there was no common set of lymphoma driver mutations in these two populations. The significance of the *PRB4* mutation is uncertain; but given it was present at low VAF and not previously reported in lymphoma, it is unlikely of significance in AITL. Further WES studies of more AITL cases with co‐existing EBV+ B cells would be of interest to further study clonal relationships (if any) between the two components.

Mouse models of AITL have been developed over the years that recapitulate some or most features of AITL and have been recently reviewed [28]. Arguably the two that recapitulate AITL most faithfully incorporate combined *TET2* knockout with expression of the RHO G17V in CD4^+^ T cells [16, 29]. Both models develop Tfh phenotype systemic lymphoma and provide valuable insight into the pathogenesis of AITL. However, neither model includes an EBV+ B‐cell component making study of the interplay between EBV+ B cells and neoplastic T cells in AITL difficult. Thus, our PDX data may be of particular interest. Further studies are needed to determine how a B‐cell component in the TME could support the neoplastic T cells.

In summary, AITL may develop EBV+ lymphoproliferative disorders during disease evolution. A PDX mouse model of AITL was established with coexisting T‐ and B‐cell compartments, and the engrafted T cells represent primary tumor cells. Growth characteristics suggest the possibility of an interaction between AITL cells and B cells in the microenvironment. This model was capable of therapeutic evaluation of agents targeting both malignant T cells and B cells simultaneously. The in vivo data support further clinical investigation of using elotuzumab or romidepsin in combination with rituximab in AITL containing EBV+ B‐cell disease.

## AUTHOR CONTRIBUTIONS

**Xiaoxian Zhao** and **Eric D. Hsi** are the principal investigators and take primary responsibility for the manuscript. **Xiaoxian Zhao**; **Juraj Bodo; Lisa Durkin**; and **Daniel J. Lindner** performed laboratory work and data analysis for this study. **Deepa Jagadeesh** and **Sarah L. Ondrejka** provided materials. **Xiaoxian Zhao** and **Eric D. Hsi** coordinated the research. **Xiaoxian Zhao; Deepa Jagadeesh; Juraj Bodo**; **Daniel J. Lindner; Sarah L. Ondrejka**; and **Eric D. Hsi** contributed to the experimental design and writing of the manuscript.

## CONFLICT OF INTEREST STATEMENT

The authors declare they have no conflicts of interest.

## ETHICS STATEMENT

Animal experiments using mice were performed in accordance with the recommendations in Guide for the Care and Use of Laboratory Animals of the National Institutes of Health, and conducted under a protocol approved by Cleveland Clinic Institutional Animal Care and Use Committee. Studies were supported in part by the Case Comprehensive Cancer Center Athymic Animal and Preclinical Therapeutics Shared Resource and NCI core grant 5 P30 CA043703‐32.

## PATIENT CONSENT STATEMENT

The authors have confirmed patient consent statement is not needed for this submission.

## CLINICAL TRIAL REGISTRATION

The authors have confirmed clinical trial registration is not needed for this submission.

## Supporting information



Supporting Information

Supporting Information

## Data Availability

Study data are available upon request if in line with ethical and legal permissions.

## References

[1] Advani RH , Skrypets T , Civallero M , Shustov AR , Hitz F , Dlouhy I , et al. Outcomes and prognostic factors in angioimmunoblastic T‐cell lymphoma: final report from the international T‐cell Project. Blood. 2021;138(3):213–220. 10.1182/blood.2020010387 34292324 PMC8493974

[2] Ellin F , Landstrom J , Jerkeman M , Relander T . Real‐world data on prognostic factors and treatment in peripheral T‐cell lymphomas: a study from the Swedish Lymphoma Registry. Blood. 2014;124(10):1570–1577. 10.1182/blood-2014-04-573089 25006130

[3] Hsi ED , Steinle R , Balasa B , Szmania S , Draksharapu A , Shum BP , et al. CS1, a potential new therapeutic antibody target for the treatment of multiple myeloma. Clin Cancer Res. 2008;14(9):2775–2784. 10.1158/1078-0432.CCR-07-4246 18451245 PMC4433038

[4] Hsi ED , Steinle R , Balasa B , Rice AG , Ko YH , Afar DE . CS1 is expressed in nasal type NK/T cell lymphomas and angioimmunoblastic T‐cell lymphomas: implications for targeted therapy with elotuzumab (HuLuc63). Blood. 2008;112(11):1779.

[5] Dupuis J , Boye K , Martin N , Copie‐Bergman C , Plonquet A , Fabiani B , et al. Expression of CXCL13 by neoplastic cells in angioimmunoblastic T‐cell lymphoma (AITL): a new diagnostic marker providing evidence that AITL derives from follicular helper T cells. Am J Surg Pathol. 2006;30(4):490–494. 10.1097/00000478-200604000-00009 16625095

[6] Marafioti T , Paterson JC , Ballabio E , Chott A , Natkunam Y , Rodriguez‐Justo M , et al. The inducible T‐cell co‐stimulator molecule is expressed on subsets of T cells and is a new marker of lymphomas of T follicular helper cell‐derivation. Haematologica. 2010;95(3):432–439. 10.3324/haematol.2009.010991 20207847 PMC2833073

[7] Dunleavy K , Wilson WH , Jaffe ES . Angioimmunoblastic T cell lymphoma: pathobiological insights and clinical implications. Curr Opin Hematol. 2007;14(4):348–353. 10.1097/MOH.0b013e328186ffbf 17534160

[8] Lunning MA , Vose JM . Angioimmunoblastic T‐cell lymphoma: the many‐faced lymphoma. Blood. 2017;129(9):1095–1102. 10.1182/blood-2016-09-692541 28115369

[9] Dobay MP , Lemonnier F , Missiaglia E , Bastard C , Vallois D , Jais JP , et al. Integrative clinicopathological and molecular analyses of angioimmunoblastic T‐cell lymphoma and other nodal lymphomas of follicular helper T‐cell origin. Haematologica. 2017;102(4):e148–e151. 10.3324/haematol.2016.158428 28082343 PMC5395128

[10] Tokunaga T , Shimada K , Yamamoto K , Chihara D , Ichihashi T , Oshima R , et al. Retrospective analysis of prognostic factors for angioimmunoblastic T‐cell lymphoma: a multicenter cooperative study in Japan. Blood. 2012;119(12):2837–2843. 10.1182/blood-2011-08-374371 22308294

[11] Hsi ED , Horwitz SM , Carson KR , Pinter‐Brown LC , Rosen ST , Pro B , et al. Analysis of peripheral T‐cell lymphoma diagnostic workup in the United States. Clin Lymphoma Myeloma Leuk. 2017;17(4):193–200. 10.1016/j.clml.2016.10.001 28209473

[12] Abruzzo LV , Schmidt K , Weiss LM , Jaffe ES , Medeiros LJ , Sander CA , et al. B‐cell lymphoma after angioimmunoblastic lymphadenopathy: a case with oligoclonal gene rearrangements associated with Epstein–Barr virus. Blood. 1993;82(1):241–246. https://www.ncbi.nlm.nih.gov/pubmed/8391875 8391875

[13] Attygalle A , Al‐Jehani R , Diss TC , Munson P , Liu H , Du MQ , et al. Neoplastic T cells in angioimmunoblastic T‐cell lymphoma express CD10. Blood. 2002;99(2):627–633. 10.1182/blood.v99.2.627 11781247

[14] Zettl A , Lee SS , Rudiger T , Starostik P , Marino M , Kirchner T , et al. Epstein–Barr virus‐associated B‐cell lymphoproliferative disorders in angloimmunoblastic T‐cell lymphoma and peripheral T‐cell lymphoma, unspecified. Am J Clin Pathol. 2002;117(3):368–379. 10.1309/6UTX-GVC0-12ND-JJEU 11888076

[15] Couronne L , Bastard C , Bernard OA . TET2 and DNMT3A mutations in human T‐cell lymphoma. N Engl J Med. 2012;366(1):95–96. 10.1056/NEJMc1111708 22216861

[16] Cortes JR , Ambesi‐Impiombato A , Couronne L , Quinn SA , Kim CS , da Silva Almeida AC , et al. RHOA G17V induces T follicular helper cell specification and promotes lymphomagenesis. Cancer Cell. 2018;33(2):259–273.e7. 10.1016/j.ccell.2018.01.001 29398449 PMC5811310

[17] Shi J , Bodo J , Zhao X , et al. SLAMF7 (CD319/CS1) is expressed in plasmablastic lymphoma and is a potential diagnostic marker and therapeutic target. Br J Haematol. 2019;185(1):145–147. 10.1111/bjh.15393 29785767

[18] Sokol K , Kartan S , Johnson WT , Alpdogan O , Nikbakht N , Haverkos BM , et al. Extreme peripheral blood plasmacytosis mimicking plasma cell leukemia as a presenting feature of angioimmunoblastic T‐cell lymphoma (AITL). Front Oncol. 2019;9:509. 10.3389/fonc.2019.00509 31263679 PMC6584846

[19] Chihara D , Fanale MA , Miranda RN , Noorani M , Westin JR , Nastoupil LJ , et al. The survival outcome of patients with relapsed/refractory peripheral T‐cell lymphoma‐not otherwise specified and angioimmunoblastic T‐cell lymphoma. Br J Haematol. 2017;176(5):750–758. 10.1111/bjh.14477 27983760 PMC5836501

[20] Kasahara H , Kakimoto T , Saito H , Akuta K , Yamamoto K , Ujiie H , et al. Successful treatment with rituximab for angioimmunoblastic T‐cell lymphoma. Leuk Res Rep. 2013;2(1):36–38. 10.1016/j.lrr.2013.03.001 24371775 PMC3850383

[21] de Leval L , Rickman DS , Thielen C , Reynies Ad , Huang YL , Delsol G , et al. The gene expression profile of nodal peripheral T‐cell lymphoma demonstrates a molecular link between angioimmunoblastic T‐cell lymphoma (AITL) and follicular helper T (TFH) cells. Blood. 2007;109(11):4952–4963. 10.1182/blood-2006-10-055145 17284527

[22] Rolf J , Bell SE , Kovesdi D , Janas ML , Soond DR , Webb LM , et al. Phosphoinositide 3‐kinase activity in T cells regulates the magnitude of the germinal center reaction. J Immunol. 2010;185(7):4042–4052. 10.4049/jimmunol.1001730 20826752

[23] Baumjohann D , Preite S , Reboldi A , Ronchi F , Ansel KM , Lanzavecchia A , et al. Persistent antigen and germinal center B cells sustain T follicular helper cell responses and phenotype. Immunity. 2013;38(3):596–605. 10.1016/j.immuni.2012.11.020 23499493

[24] Fujisawa M , Nguyen TB , Abe Y , Suehara Y , Fukumoto K , Suma S , et al. Clonal germinal center B cells function as a niche for T‐cell lymphoma. Blood. 2022;140(18):1937–1950. 10.1182/blood.2022015451 35921527 PMC10653021

[25] Delfau‐Larue MH , de Leval L , Joly B , Plonquet A , Challine D , Parrens M , et al. Targeting intratumoral B cells with rituximab in addition to CHOP in angioimmunoblastic T‐cell lymphoma. A clinicobiological study of the GELA. Haematologica. 2012;97(10):1594–1602. 10.3324/haematol.2011.061507 22371178 PMC3487562

[26] Li C , HU H , Lei T , Yu H , Chen X , Peng S , et al. Updated results of a prospective study: rituximab and lenalidomide plus chidamide (RLC) for patients with relapsed/refractory angioimmunoblastic T‐cell lymphoma. Blood. 2023;142(Supplement 1):6221–6221. 10.1182/blood-2023-184830

[27] Nkosi D , Allbee AW , Rothberg PG , Friedberg JW , Evans AG . Common clonal origin of three distinct hematopoietic neoplasms in a single patient: B‐cell lymphoma, T‐cell lymphoma, and polycythemia vera. Cold Spring Harb Mol Case Stud. 2024;9(4):a006313. 10.1101/mcs.a006313 38199781 PMC10815289

[28] Mhaidly R , Krug A , Gaulard P , Lemonnier F , Ricci JE , Verhoeyen E . New preclinical models for angioimmunoblastic T‐cell lymphoma: filling the GAP. Oncogenesis. 2020;9(8):73. 10.1038/s41389-020-00259-x 32796826 PMC7427806

[29] Ng SY , Brown L , Stevenson K , deSouza T , Aster JC , Louissaint A Jr , et al. RhoA G17V is sufficient to induce autoimmunity and promotes T‐cell lymphomagenesis in mice. Blood. 2018;132(9):935–947. 10.1182/blood-2017-11-818617 29769264 PMC10251505

