# Supplementary material for: Angioimmunoblastic T‐cell lymphoma: Characterization of clonal T and B cells and a patient‐derived xenograft study of coexisting T‐ and B‐cell proliferation
Source: EJHaem. 2025 Jan 28;6(1):e1080. doi: 10.1002/jha2.1080 (PMC11773159; doi:10.1002/jha2.1080)

Supplemental Figure 1

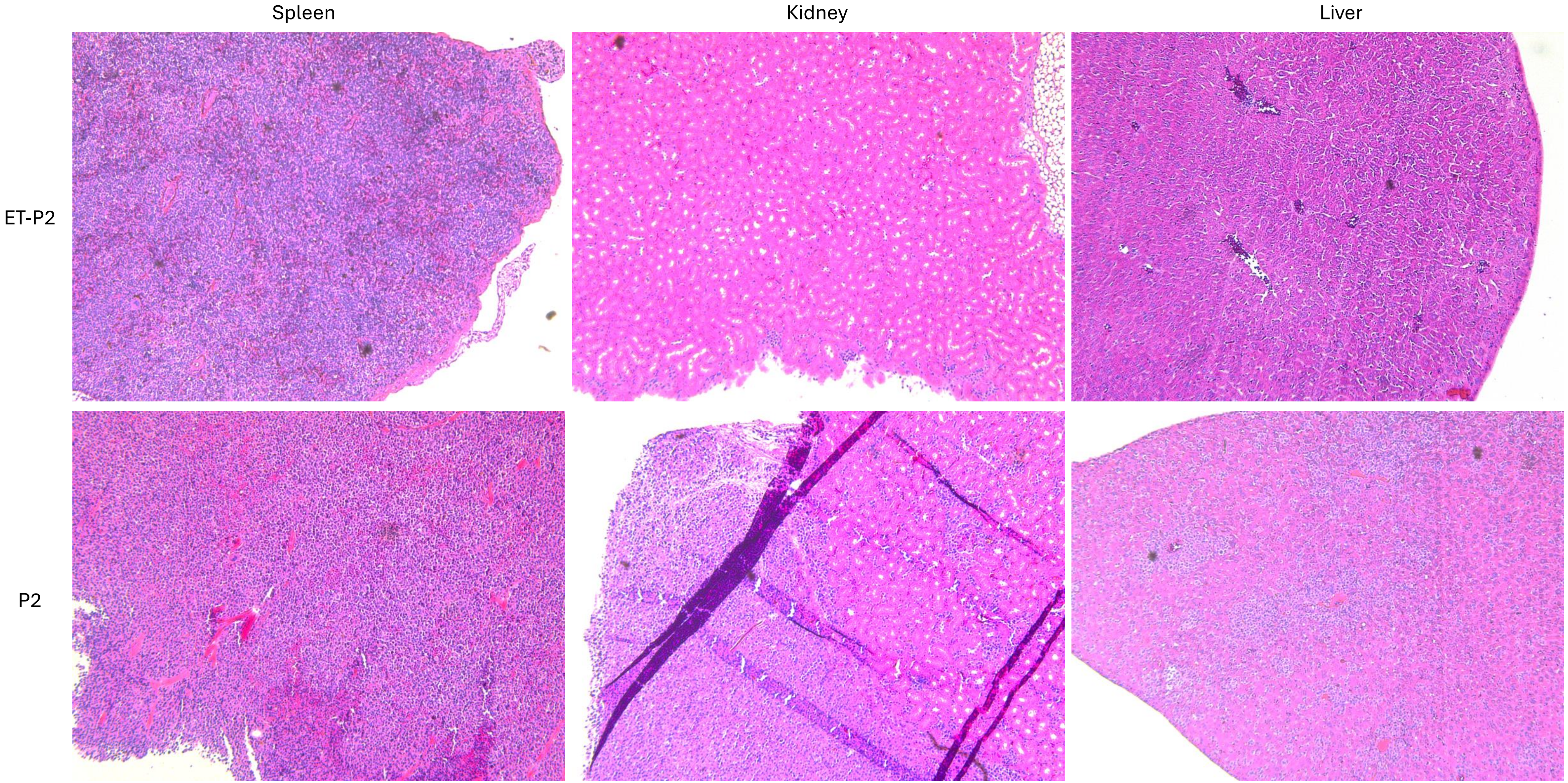

Supplemental Figure 2

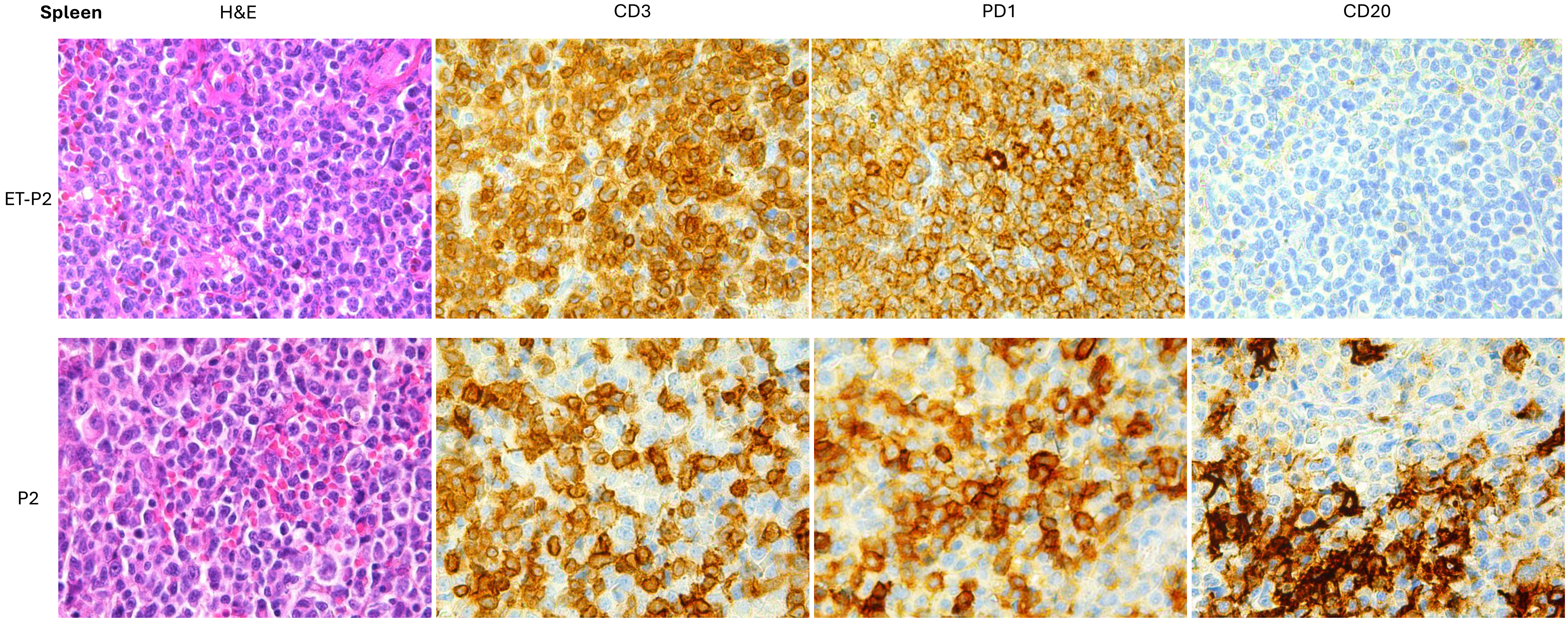

Supplemental Figure 2

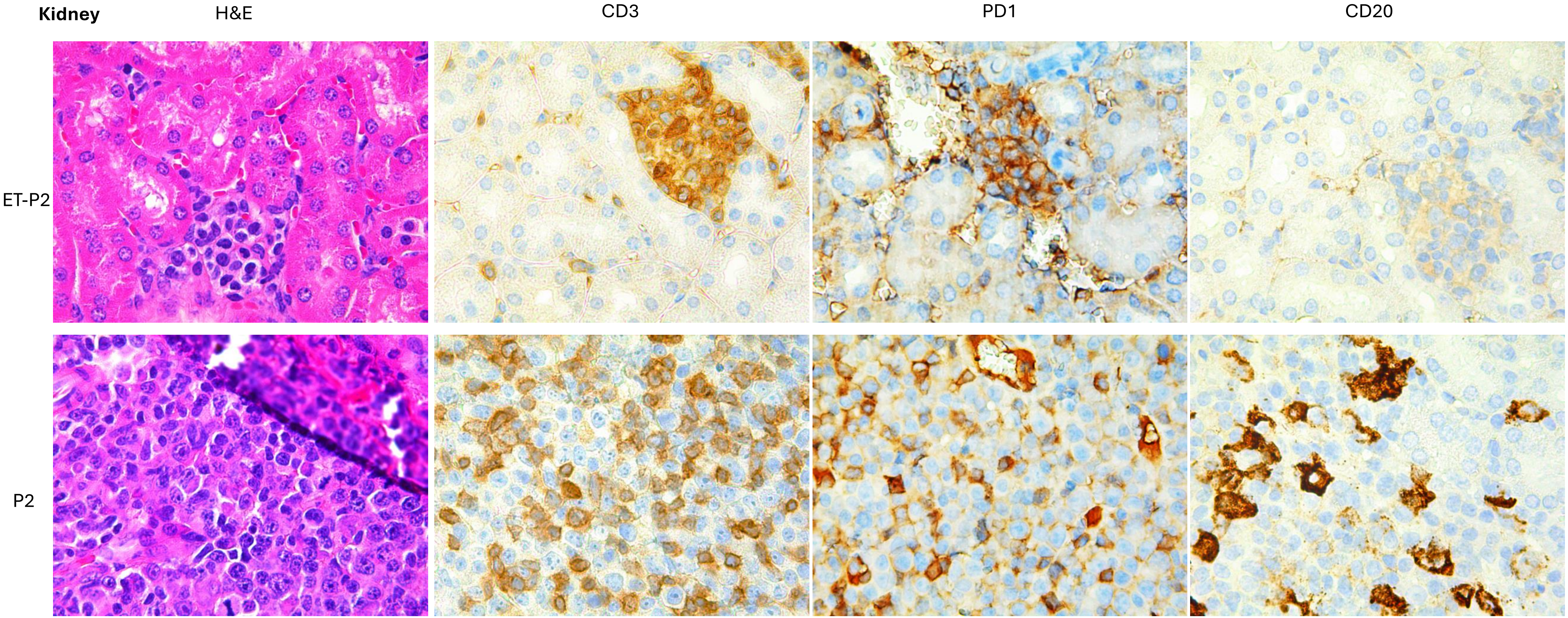

Supplemental Figure 2

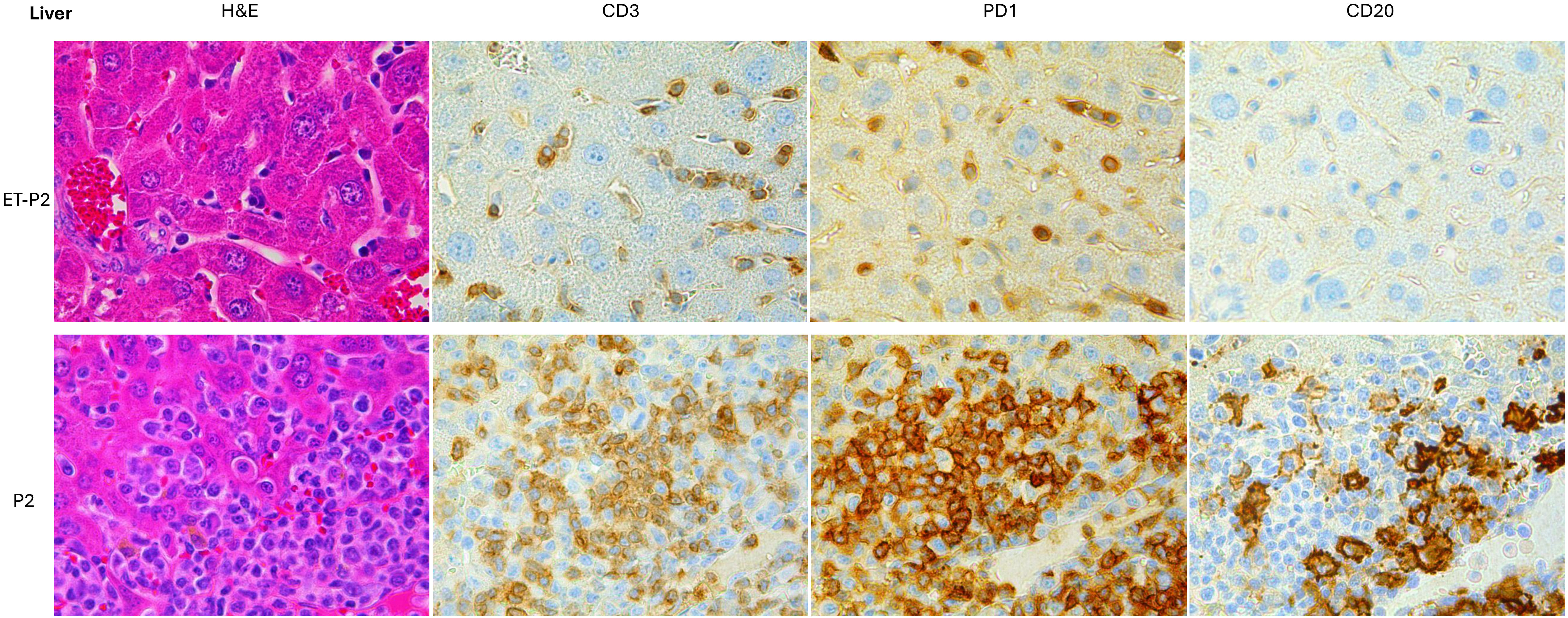

Supplemental Figure 3

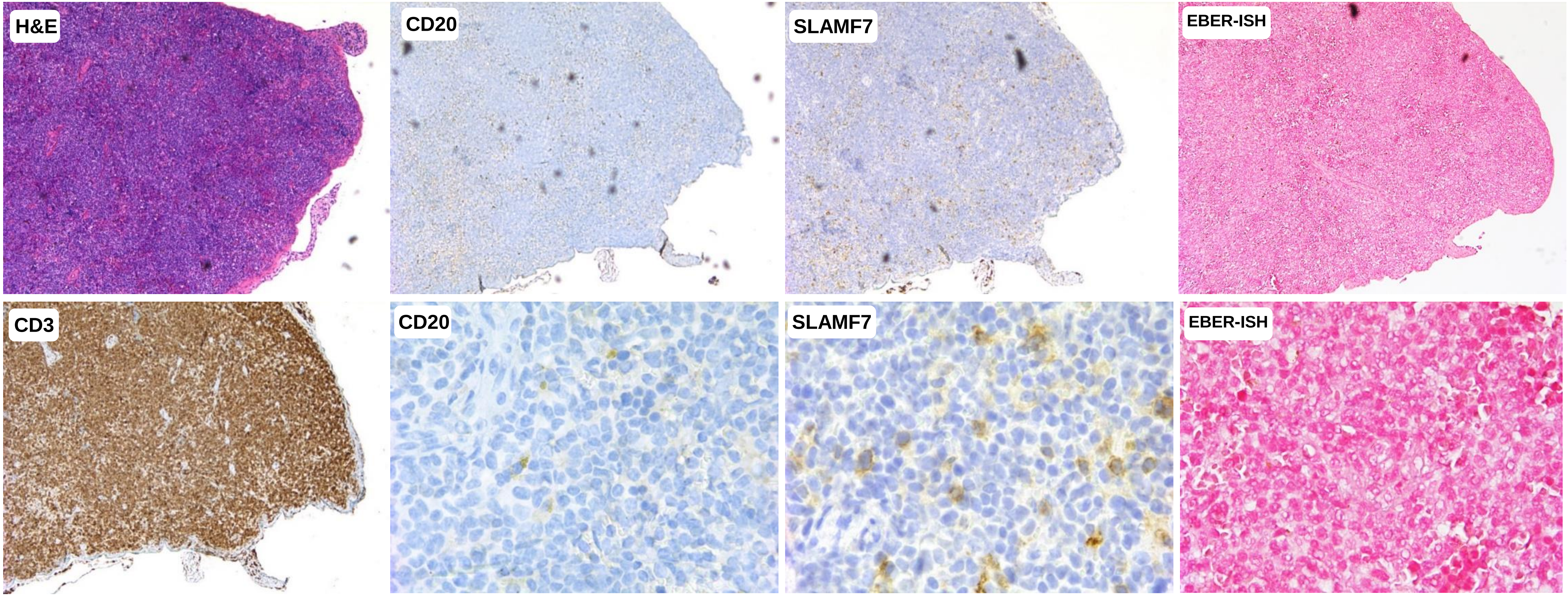

Supplemental Figure 4

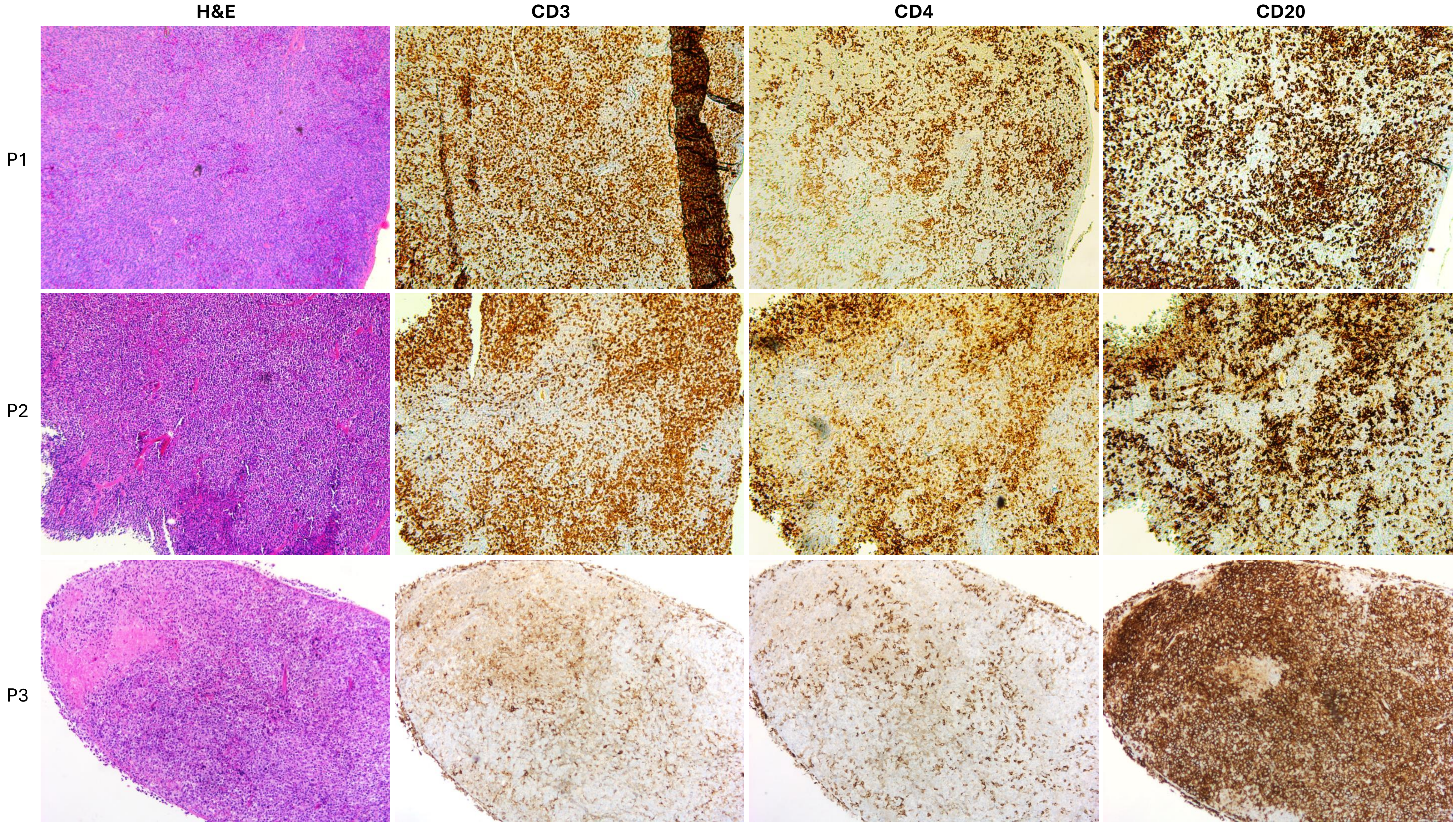

Supplemental Figure 5

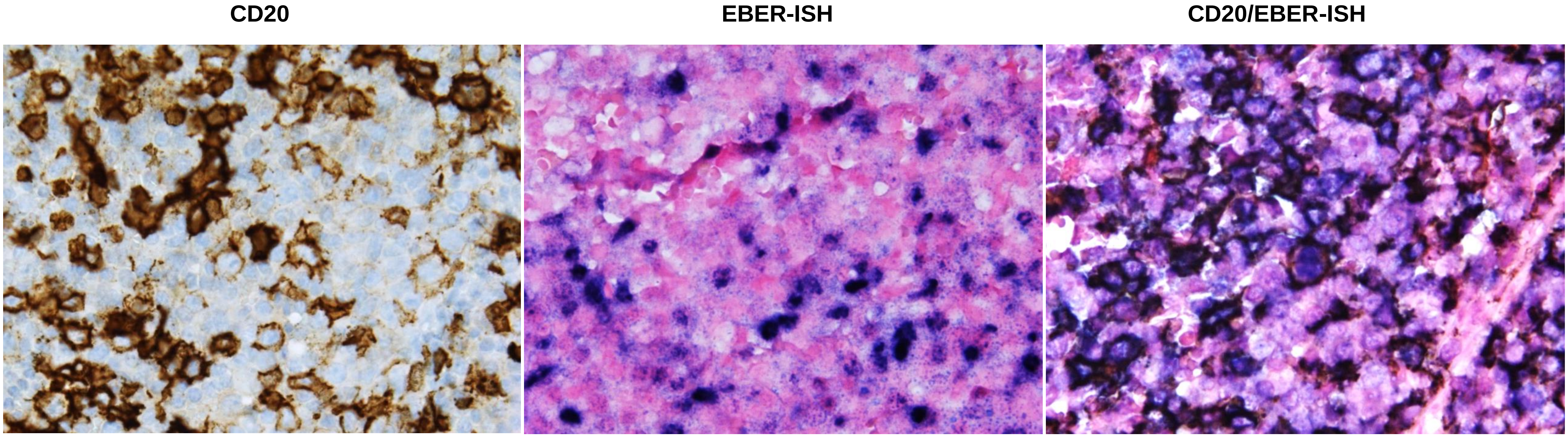

Supplemental Figure 6

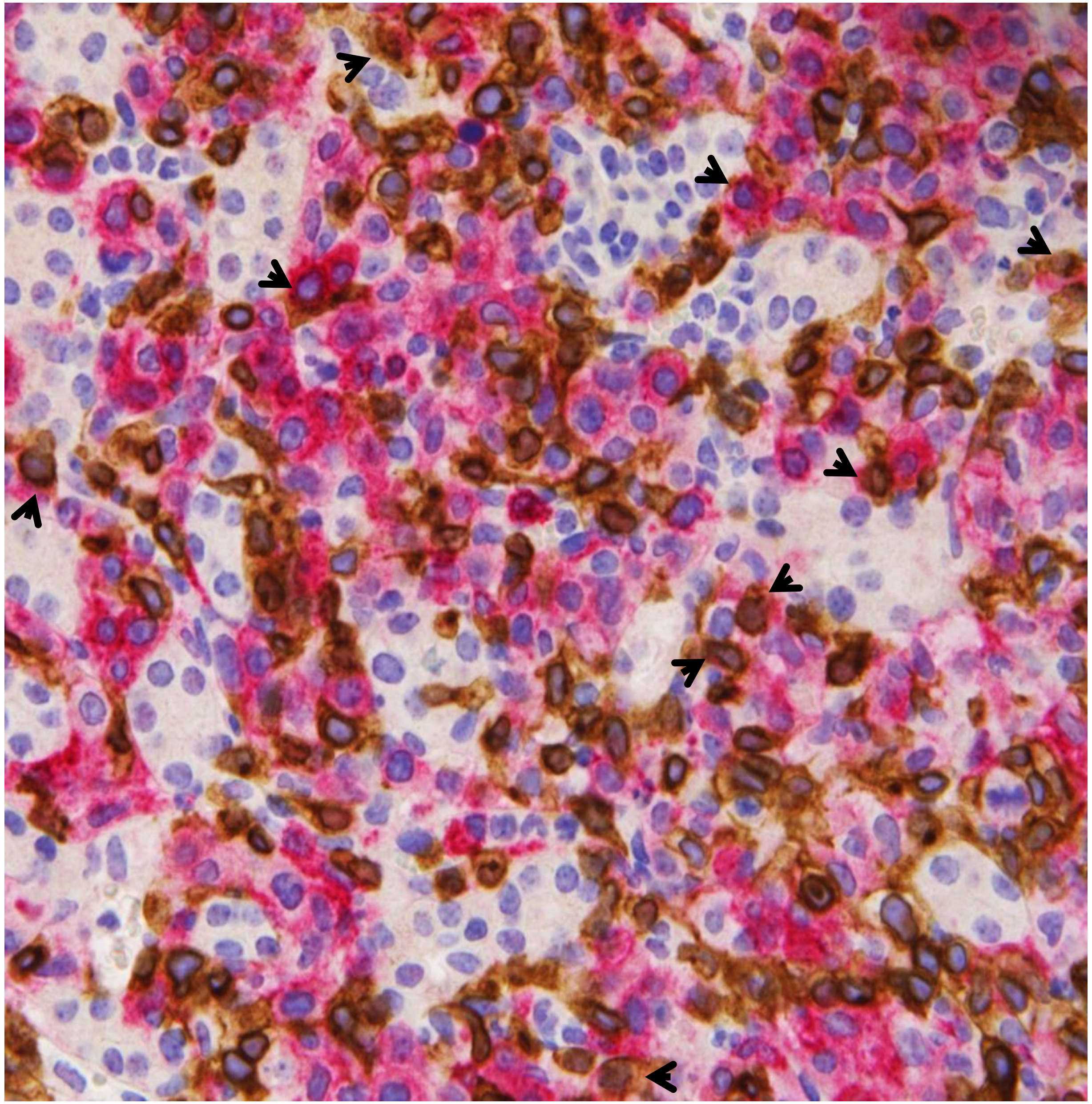

Supplemental Figure 7

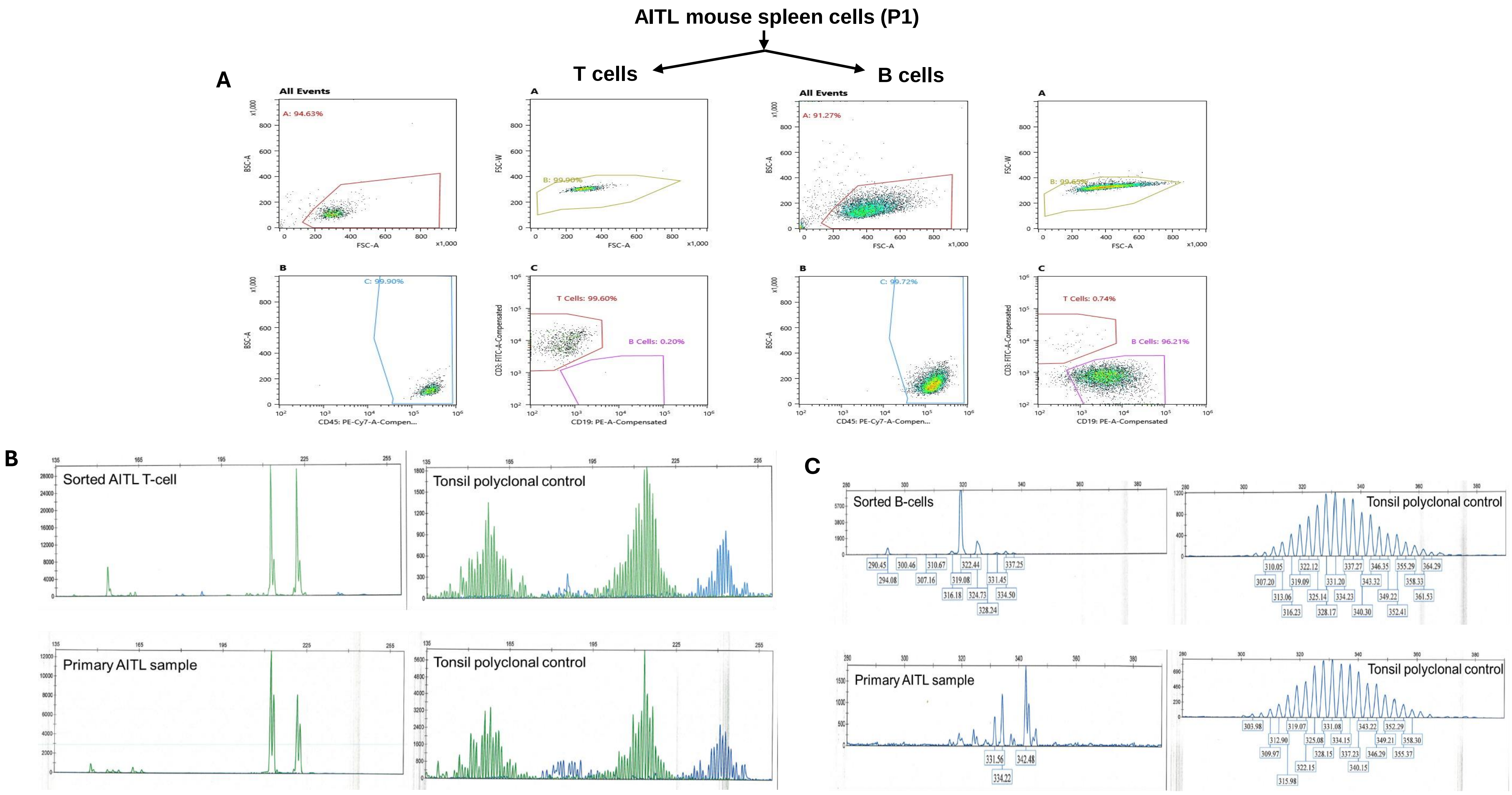

Supplemental Figure 8

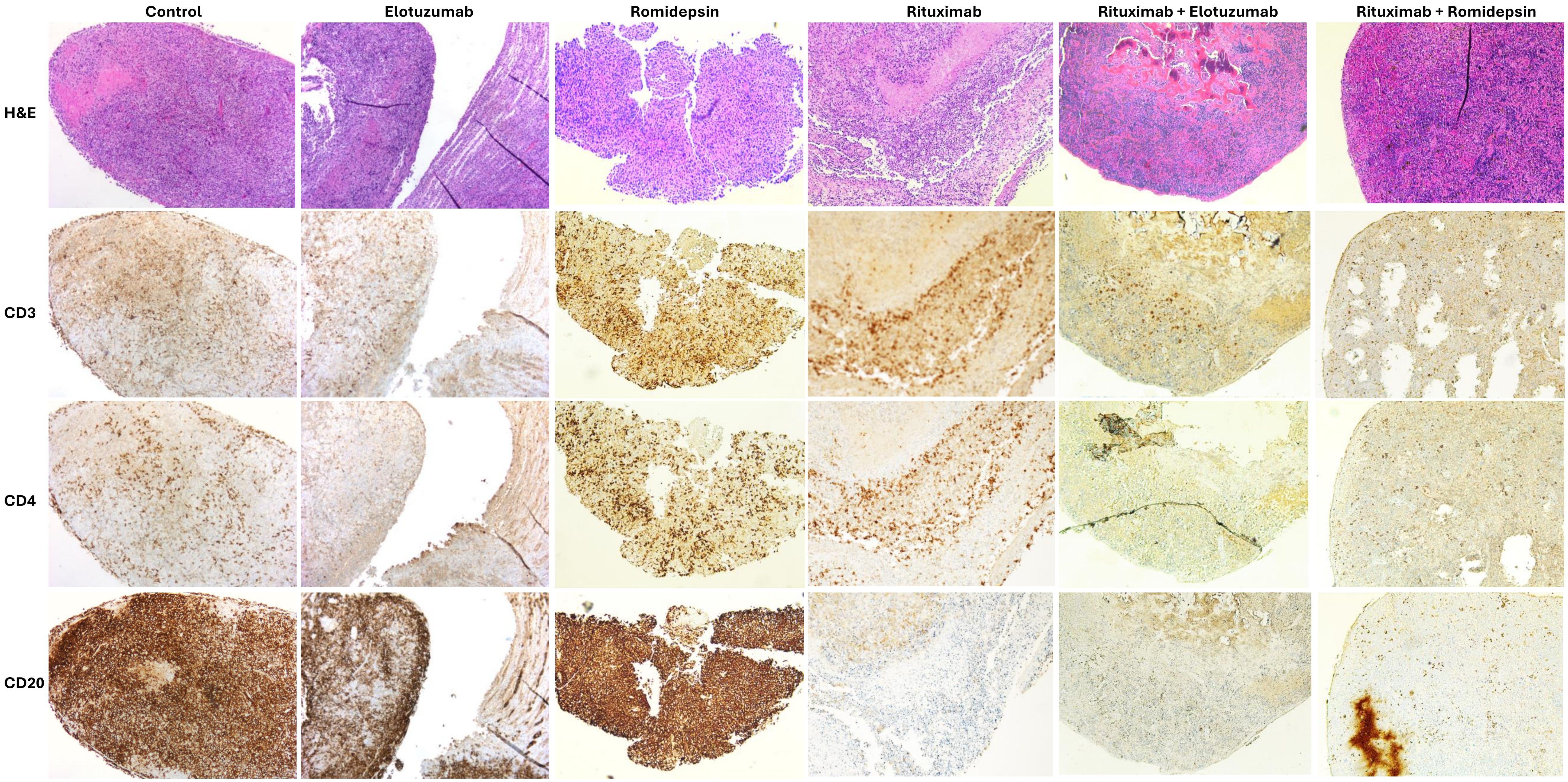

Supplement: Supplementary file 1 — Supporting Information [file JHA2-6-e1080-s001.pdf]
