# Supplementary material for: Angioimmunoblastic T‐cell lymphoma: Characterization of clonal T and B cells and a patient‐derived xenograft study of coexisting T‐ and B‐cell proliferation
Source: EJHaem. 2025 Jan 28;6(1):e1080. doi: 10.1002/jha2.1080 (PMC11773159; doi:10.1002/jha2.1080)
Supplement: Supplementary file 2 — Supporting Information [file JHA2-6-e1080-s002.docx]

**Supplemental Information**

**Angioimmunoblastic T-cell Lymphoma: Characterization of Clonal T- and B-cells and a Patient Derived Xenograft study of Coexisting T- and B-cell Proliferation**

Xiaoxian Zhao^1,2^, Deepa Jagadeesh^3^, Juraj Bodo^1^, Lisa Durkin^1^, Daniel J. Lindner^3^, Sarah L. Ondrejka^1^, Eric D. Hsi^1,2*­­­­^

^1^Pathology and Lab Medicine Institute, Cleveland Clinic, Cleveland OH, ^2^Wake Forest University School of Medicine, Winston Salem, NC, ^3^Taussig Cancer Center, Cleveland Clinic, Cleveland OH

*Current affiliation: Department of Laboratory Medicine and Pathology, Mayo Clinic, Rochester, MN

**Materials and Methods**

***Immunophenotyping***

Cell surface markers were analyzed by flow cytometry (MACSQuant Analyzer 10, Miltenyi Biotech). Peripheral blood samples, bone marrow cells or isolated tissue cells were stained with indicated antibodies for 20 minutes at room temperature and washed with PBS for analysis.

***Immunohistochemistry (IHC) and CISH-EBER***

Harvested mouse organs were fixed, and paraffin embedded. Tissue sections were stained with hematoxylin and eosin (H&E) and IHC (antibodies listed in supplemental Table 1). CISH-EBER assay was performed using automated stainers (Ventana Benchmark Ultra). Double staining for CD3 (Clone LN10, Leica Biosystems)/SLAMF7 and CD20/SLAMF7 was performed in Bond Max autostainer and detected with Bond Polymer Refine Detection and Bond Polymer Refine Red Detection, respectively. For CD20/EBER ISH double staining, EBER ISH was first run in the clinical IHC lab of Cleveland Clinic, followed by CD20 IHC on Ventana automated stainer.

***Enrichment / sorting of engrafted tumor cells and DNA extraction***

Cells isolated from primary AITL engrafted mouse spleen (passage 1, P1) were used for 1) Enrichment of T cells (RosetteSep^TM^ Human T-cell Enrichment Kit, STEMCELL Technologies). Enriched T-cells were inoculated into NSG mice in parallel with P1 mouse spleen cells without enrichment. 2) Sorting for both T cells and B cells with SH800S Cell Sorter (Sony Biotechnology). CD15+ neutrophils were sorted from paired patient normal peripheral blood. Genomic DNA was extracted from sorted cells (QIAamp DNA Mini Kit, Qiagen).

***T-cell and B-cell clonality analysis***

PCR based clonality assays for T-cell and B-cell with BIOMED-2 Primers (Invivo scribe, San Diego, CA) were performed in the clinical laboratories of Cleveland Clinic following standard clinical protocols.

***Whole Exome Sequencing (WES)***

Genomic DNA from sorted T, B cells and paired normal neutrophils were used for WES which was performed and analyzed at Otogenetics Corporation (Atlanta, GA) with paired-end sequencing 2 x 100 bp using Hiseq2500 platform (Illumina, San Diego, CA) and Agilent Human exome V5 (51 Mb) capture kit designed by Agilent technologies (Mississauga, Ont). The human genome reference sequence hg19 was used to align reads. Analysis was focused on comparison of variants between tumor and paired normal control.

**Supplemental Figure Legends**

**Supplemental figure 1**: Infiltration of AITL tumor cells in NSG mouse organs (H&E staining). ET-P2: passage 2 mouse injected with enriched T-cell from first passage AITL cells engrafted mouse spleen; P2: passage 2 mouse injected with cells from first passage AITL cells engrafted mouse spleen. The ET-P2 spleen shows diffuse infiltration. The kidney and liver tissues show intact architecture with, at most, a sparse interstitial infiltrate without tumor forming lesions. However, the P2 tissues show areas of architectural effacement (spleen, kidney) or microscopic large aggregates/tumorlets (liver).

**Supplemental figure 2**: H&E and IHC staining of AITL lymphoma engrafted cells in NSG mouse organs. The ET-P2 spleen is infiltrated by T-cell lymphoma expressing PD1 without B-cells. The P2 spleen shows both components. The ET-P2 kidney shows only scattered collections of Tfh cells but no B-cells. In contrast the P2 kidney has a dense infiltrate with both components. The ET-P2 liver contains only rare interstitial T-cells expressing PD1 without B-cells. The P2 liver shows lymphoma infiltration with both Tfh T-cells and B-cells.

**Supplemental figure 3**: CD3 and weak partial SLAMF7 expression on T-cells but lack of CD20 and EBER expression in engrafted ET-P2 mouse spleen.

**Supplemental figure 4**: HE and IHC staining of human AITL T & B-cell markers in passages 1, 2 and 3 (P1, P2 and P3) engrafted mice spleen tissue. Which showed an observable increase of B-cell in P3 mouse spleen compared to P1 & P2 samples.

**Supplemental figure 5**. CD20 and EBER double staining of AITL cell engrafted mouse spleen. Single staining of CD20 (left panel) and EBER-ISH (middle panel) show the positive B-cells and EBER, respectively. Double staining shows the positive staining of EBER in CD20+ cells.

**Supplemental figure 6**. Double staining of AITL cell engrafted mouse spleen. Arrows indicate a subset of T-cells coexpressed SLAMF7 (red)/CD3 (brown)

**Supplemental figure 7**. **A**: Cell-type enrichment by flow cytometry-based sorting (P1 mouse spleen cells) showed high T or B-cell purity prior to inoculation. **B & C**: T cell and B-cell receptor gene rearrangement of the sorted T and B-cells. The monoclonal gene rearrangement pattern for sorted T-cells is identical to the primary sample, consistent with clonal identity between the two samples. B-cell clonality was detected in the sorted B-cells but was not detected in the primary sample, likely due to low percentage of EBV+ B-cells in the primary tissue (<2%).

**Supplemental figure 8**: Representative HE and IHC staining of human AITL T & B-cell markers in mice spleen tissue of each treated cohort, in which elotuzumab or romidepsin alone showed similar staining pattern compared to control samples, while rituximab caused a decrease of CD20+ cells but minor effects on CD3+ and CD4+ T cells. Treatment with two agents combined groups resulted in reduction of both T and B-cell compared to control and single agent treated cohort.

**Supplemental table 1:**

**IHC antibodies**

| **Antibody** | **CD3** | **CD4** | **CD20** | **BCL-6** | **PD1** | **SLAMF7** | **Cleaved PARP** |
| --- | --- | --- | --- | --- | --- | --- | --- |
| **Vendor** | Ventana | Ventana | Ventana | Dako | Abcam | LS Biosciences | Cell Signaling technology |
| **Clone** | 2GV6 | SP35 | L26 | PG-B6p | NAT105 | OTI3B3 | D64E10 |
